# Procedures for the Quantification of Whole-Tissue Immunofluorescence Images Obtained at Single-Cell Resolution during Murine Tubular Organ Development

**DOI:** 10.1371/journal.pone.0135343

**Published:** 2015-08-10

**Authors:** Tsuyoshi Hirashima, Taiji Adachi

**Affiliations:** Institute for Frontier Medical Sciences, Kyoto University, Kyoto, Japan; University of Hull, UNITED KINGDOM

## Abstract

Whole-tissue quantification at single-cell resolution has become an inevitable approach for further quantitative understanding of morphogenesis in organ development. The feasibility of the approach has been dramatically increased by recent technological improvements in optical tissue clearing and microscopy. However, the series of procedures required for this approach to lead to successful whole-tissue quantification is far from developed. To provide the appropriate procedure, we here show tips for each critical step of the entire process, including fixation for immunofluorescence, optical clearing, and digital image processing, using developing murine internal organs such as epididymis, kidney, and lung as an example. Through comparison of fixative solutions and of clearing methods, we found optimal conditions to achieve clearer deep-tissue imaging of specific immunolabeled targets and explain what methods result in vivid volume imaging. In addition, we demonstrated that three-dimensional digital image processing after optical clearing produces objective quantitative data for the whole-tissue analysis, focusing on the spatial distribution of mitotic cells in the epididymal tubule. The procedure for the whole-tissue quantification shown in this article should contribute to systematic measurements of cellular processes in developing organs, accelerating the further understanding of morphogenesis at the single cell level.

## Introduction

Beyond the genomic era, systematic observation and quantification at single-cell resolution has become a powerful approach to investigate which and how cellular processes, such as cell division or active cellular constriction, underlie morphogenesis during the embryonic development [1–3]. Employing such an approach in morphogenesis research has progressed the understanding of biological processes bridging different scales between the cell and tissue levels. Fluorescence labeling has been widely used for the detection of these cellular processes. Quantifying the intensity and spatial distribution of fluorescence signals through comprehensive observation, even in fixed tissues, leads to a trigger for the discovery of core cellular processes pertinent to the dynamic aspects of morphogenesis during organ development [4,5].

Exhaustive examination throughout developing tissues using fluorescence imaging is required to observe key morphogenetic processes at the cellular level. The lack of transparency in tissues, particularly in mammalian tissues, is a hurdle for clear detection of fluorescence signals when the observation targets exist in deep regions of the tissues. This difficulty in deep tissue imaging mainly arises from light scattering due to the high refractive index (RI) of biological tissues. One of the conventional ways to perform volume imaging is the three-dimensional (3D) computational reconstruction of images from a number of thin sections. However, this method often spoils the continuity of the tissues of interest [6]. Therefore, appropriate preparations that allow the realization of volume imaging without breaking the continuity of tissues have been demanded.

Optical clearing techniques have been extensively improved in the past few years to overcome this issue. These techniques have been shown to aid further deep tissue imaging. The goal during the early development of optical clearing techniques was to discover solutions that have an RI closer to that of biological tissue to reduce the RI mismatch. With this in mind, the celebrated clearing reagent BABB has been widely used because of its high RI, despite drawbacks like quenching of endogenous fluorescent proteins and shrinking of tissues. Clearing solutions that improve those drawbacks, for example, Sca*l*e and SeeDB have recently been developed [7–10]. Further improvements have been made under the concept of the removal of lipids from tissues, because the lipid components are major source of light scattering and obstruct the penetration of antibodies [11–14]. Combining these optical clearing methods with innovative fluorescence microscope technologies that allow rapid scanning of entire organs or organisms provides a systematic approach for clarifying protein distribution in a noninvasive manner. This approach has evolved to whole-tissue imaging at single-cell resolution. It will accelerate further understanding of morphogenesis from the viewpoint of systems biology on the organ to individual organism level [13–17].

Due to this technological revolution, whole-tissue analysis via deep tissue imaging has become an inevitable approach to quantify cellular processes of tissues in a systematic manner. Imaging and quantification in whole tissues provide accurate information on the spatial distribution of cellular processes because it preserves the structural continuity of tissues, while techniques using sliced tissues tend to lack tissue continuity. Thus, in this article, we provide an appropriate procedure for whole-tissue quantification at single-cell resolution in fluorescently labeled tissues of developing organs. The major aim of this study is to communicate tips concerning each critical step of the entire procedure for whole-tissue quantification, including immunofluorescence, optical clearing, and 3D digital processing, so that anyone who has less experience can perform the entire process.

The contents of this article are organized according to the sequence of the standard protocol for the whole-tissue quantification and cover only the critical steps described in Table 1. Throughout this article, we focus on developing murine tubular organs, such as epididymis, kidney, and lung. Although these organs are different in terms of shape and function, they have a common structure that a single-layered epithelial tubule is embedded in mesenchyme and extra-cellular matrix, enveloped by a clear border of organ. In the first section of the Results, we show typical images obtained through whole-tissue fluorescence immunolabeling for F-actin and E-cadherin in epithelial tubules. In the next section, we present a case, in which the fixative solutions significantly alter the labeling performance on pMLC immunofluorescence. This section is aimed to advise the readers to pay general attention to the choice of fixative in the process of whole-tissue quantification. Then, we compare established optical clearing methods regarding how much signal intensity can be detected in the deep region of tissues, and examine whether the combination of the established clearing methods can improve deep tissue imaging. Finally, automatic whole-tissue quantification for mitotic cells by digital image processing is demonstrated using the embryonic murine epididymal tubule.

**Table 1:** A typical protocol for whole-tissue quantification via immunofluorescence in descending order.

Operation	Comment
Fixation	Regarding optimal choice of fixative solution is discussed in this article (Fig 2)
Antigen retrieval	This is an optional step. Unnecessary heat activation should be avoided because heating the biological tissues tends to result in the destruction of tissue structure. We skipped this step in this study
Blocking and antibody reaction	Conditions for the immunolabeling should be optimized depending on samples although the standard ones are described in the section of Methods
Optical clearing	Comparison of optical clearing methods is discussed in this article (Fig 3)
Microscopic imaging	We used a conventional confocal microscope in this study
Digital image processing	An example of 3D digital image processing is shown in this article (Fig 4)

Only the fixation, optical clearing, and digital image processing steps are mentioned in the main text.

## Results

### Illustration of Whole-Tissue Fluorescence Imaging

**Fig 1:**
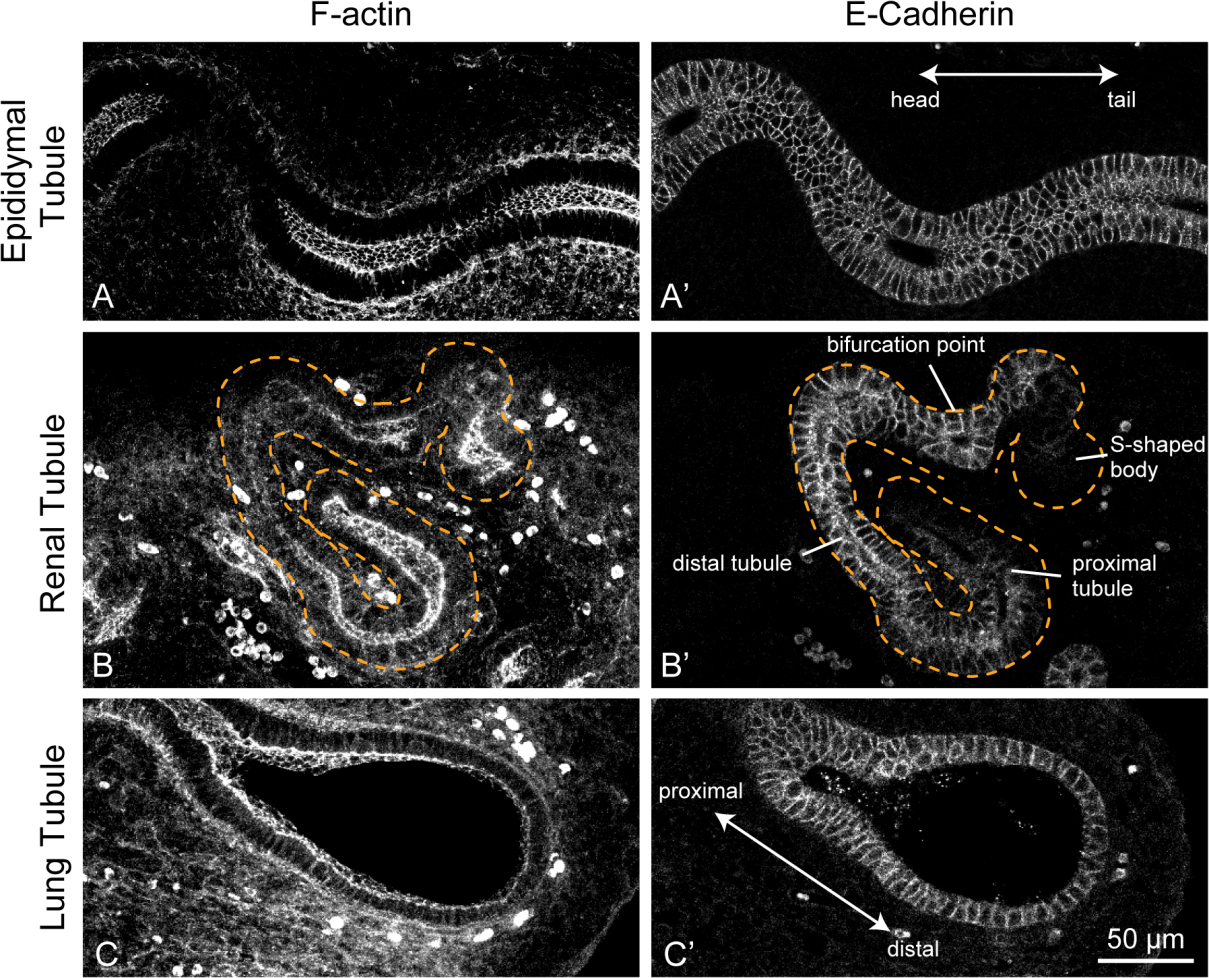
Whole-tissue fluorescence images of F-actin and E-cadherin. The spatial distribution of F-actin (left) and E-cadherin (right) during epididymal (upper), kidney (middle), and lung (bottom) development is shown. Each organ was dissected at E16.5 for epididymis and at E13.5 for both kidney and lung. Dotted orange lines in the middle row represent the tissue boundary of the epithelial renal tubule. For the images obtained with F-actin staining, a maximum intensity projection was applied over 30 μm with 2-μm intervals for the three organs. The E-cadherin images are single section views. Scale bar: 50 μm.

We first show images obtained via whole-tissue fluorescence labeling of F-actin and E-cadherin, both of which are generally expressed in epithelial cells, in the developing epididymis (E16.5), kidney (E13.5), and lung (E13.5) (Fig 1). Although these two proteins are known to be key factors for the generation of cellular physical force associated with maintaining epithelial integrity during tissue morphogenesis, the exact distribution of these proteins in whole internal organs remains elusive because the analysis has been mainly performed via slice sectioning [18–21].

Whole-tissue imaging revealed that the fluorescence signals of both F-actin and E-cadherin could be found mainly beside the cellular membranes of tubule epithelia in any organ and that the expression patterns along the tubule were characteristic of each organ. As shown in Fig 1A and 1B, 1F-actin distributes mainly at the apical junction domains of epithelial cells almost homogeneously throughout the epididymal and renal tubules. In the lung tubule, in contrast, the intensity of the F-actin signal gradually became weaker toward the more distal region of epithelial tubule (Fig 1C). Immunofluorescence signals showed that the distribution of E-cadherin was almost homogeneous throughout the tubule in the epididymis and in the lung (Fig 1A′ and 1C′). However, we found that the fluorescence signals of E-cadherin disappeared in some regions of the kidney. Indeed, the expression of E-cadherin could not be observed in the S-shaped body or in the proximal tubule of the developing kidney despite continuous connection of their epithelial tissues (Fig 1B and 1B′). This unique pattern of E-cadherin expression is consistent with the pattern obtained by slice sectioning in earlier studies [19,20]. Clearly, whole-tissue fluorescence labeling allows us to accurately explore the spatial distribution of protein localization without destroying the entire structure of fixed tissues.

### Choice of Fixative Solutions

The choice of fixative solutions is crucial to the identification of innate spatial distributions of immunolabeled proteins. In this section, we demonstrate that differences in the use of fixatives greatly influence the resulting detection of immunofluorescence signals. As an example of antigen epitopes sensitive to fixative, we introduce epitopes of phosphorylated myosin light chain (pMLC) and show the differences in the degree of detection when the internal organs were chemically fixed with 4% paraformaldehyde (PFA) and 2% trichloroacetic acid (TCA).

**Fig 2:**
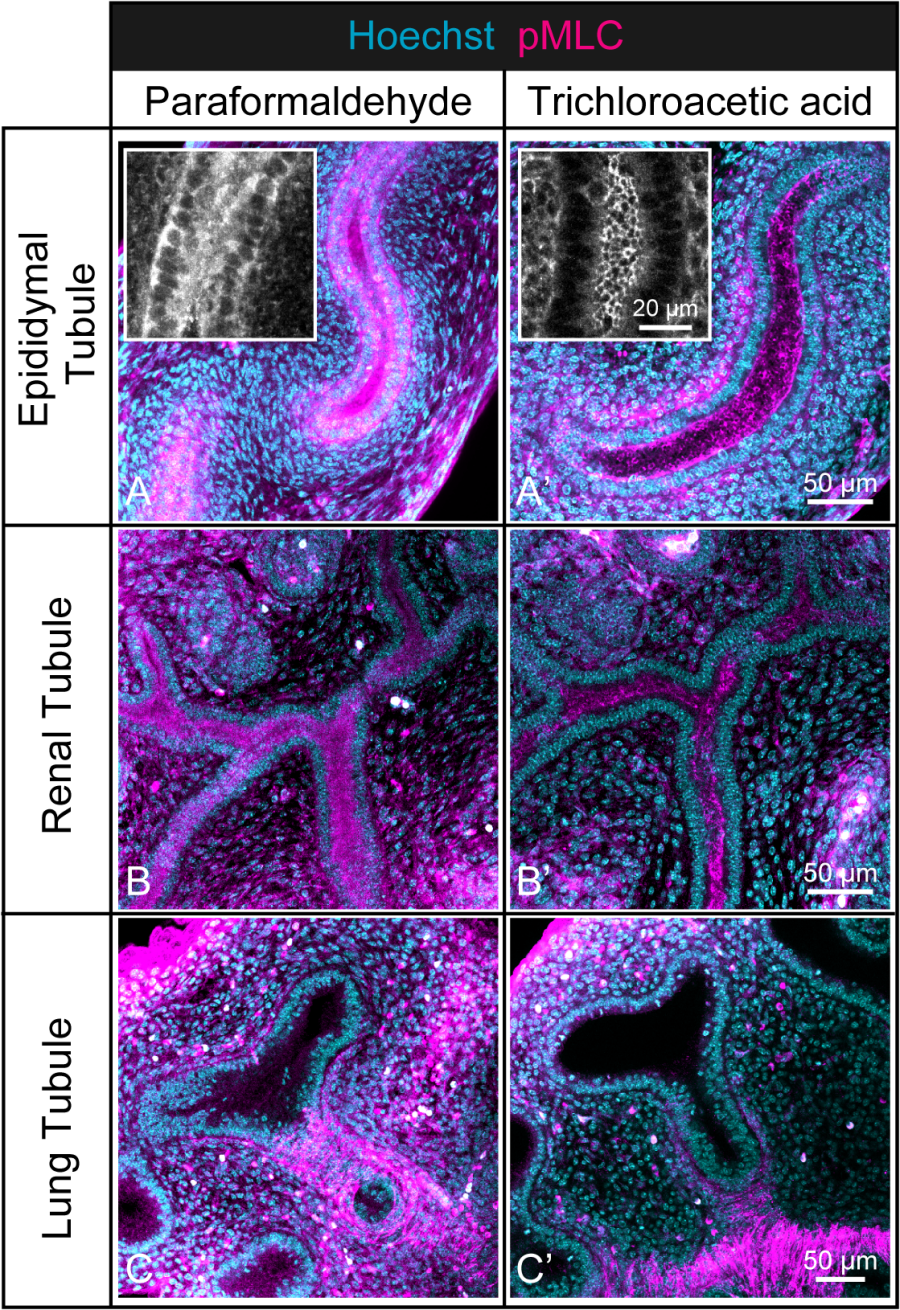
Comparison of phosphorylated myosin light chain immunofluorescence images obtained using different fixative solutions. Whole-tissue immunofluorescence of pMLC (magenta) was performed on the embryonic epididymis (upper), kidney (middle), and lung (bottom) tissues with a Hoechst dye (cyan) counter stain. Samples were chemically fixed with either 4% paraformaldehyde solution (left) or 2% trichloroacetic acid solution. Epididymis was dissected at E16.5, while the kidney and lung were dissected at E13.5. Scale bar: 50 μm. In the small windows of Fig **2A** and **A′**, only pMLC staining images were visualized, and the scale bar represents 20 μm. For the images of immunolabeled pMLC, a maximum intensity projection was applied over 33 μm with 1-μm intervals for the three organs. The images containing Hoechst dye staining are single section views.

As shown in Fig 2, the fluorescence signals of immunolabeled pMLC became much clearer with TCA fixation than with PFA fixation. TCA fixation provided detailed spatial information at single-cell resolution; the pMLC localized mainly at the apical junction of the epididymal tubule. PFA fixation resulted in blurred images (Fig 2A and 2A′). Indeed, TCA fixation contributed to good preservation of the pMLC epitopes, enabling clearer detection via whole-tissue immunofluorescence compared with when PFA fixation was used.

Treatment with the appropriate fixative reveals that the distribution of pMLC obviously varies depending on the internal organ. In the epididymis and kidney, pMLC was found to be localized almost at the apical junction of epithelial cells in the tubule (Fig 2A′ and 2B′). In the lung, pMLC distributed mainly in the mesenchyme surrounding the proximal region of the developing tubule and was arranged orthogonal to the longitudinal axis of the epithelial tubule (Fig 2C′). Interestingly, the pMLC signals completely disappeared in the distal region of the lung tubule.

### Comparison of Optical Clearing Methods

Optical tissue clearing is an essential step for vivid volume imaging at single-cell resolution in deep tissue structures. Here we chose optical clearing methods such as BABB (a mixture of benzyl-alcohol and benzyl-benzoate) [8,9], SeeDB (see deep brain) [10], CUBIC (clear, unobstructed brain imaging cocktails and computational analysis) [13,14], and PACT (passive clarity technique) [12] among published clearing methods, which satisfy criteria that the clearing process is rapid, inexpensive, and a simple immersion-based process for convenient whole-tissue volume imaging. We applied these optical clearing methods to dissected epididymis (E18.5), kidney (E16.5), and lung (E15.5) that were immunolabeled for the E-cadherin, and examined the intensity of fluorescence signals at deep region in these tissues. For each treatment with the optical clearing methods, we acquired volume images at 5-μm intervals with a confocal microscope and measured the mean intensity of the fluorescence signals for E-cadherin within an image window.

**Fig 3:**
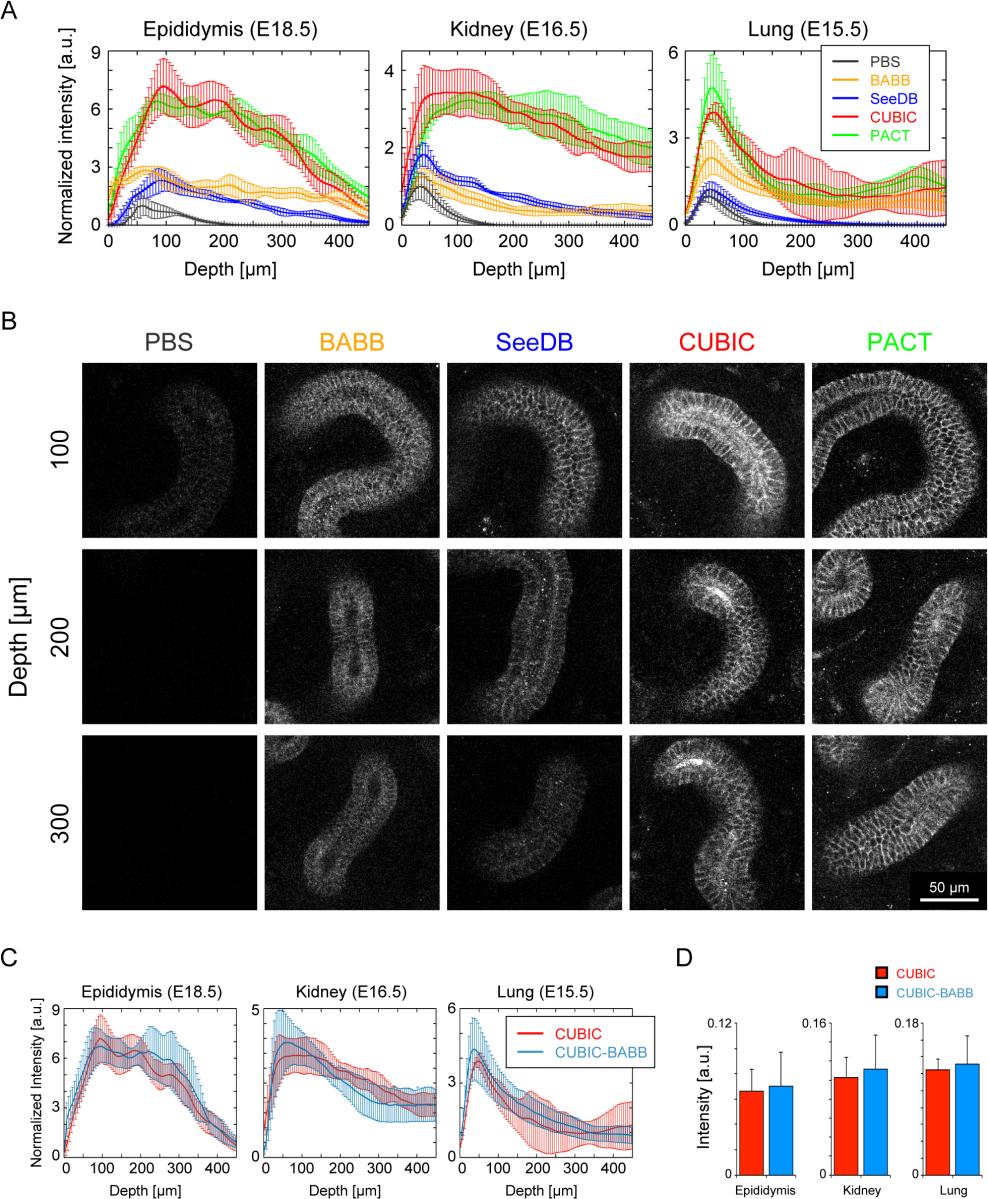
Comparison of immunofluorescence for E-cadherin among optical clearing methods. Different optical clearing methods, including BABB, SeeDB, CUBIC, and PACT, were applied to the embryonic epididymis (E18.5), kidney (E16.5), and lung (E15.5), each of which was immunolabeled with E-cadherin. PBS was used as a control solution. The color represents the optical clearing method. **(A)** Plot of the mean E-cadherin immunofluorescence intensity against the depth from the bottom of tissues embedded onto the dish. The mean intensity was normalized to the maximum intensity of PBS-treated samples (black) in each organ. *n* = 8. The error bars represent the standard deviation (s.d.). **(B)** Representative images of E-cadherin immunofluorescence in the epididymis are shown for each depth from the bottom of the tissue (row) and for each optical clearing method (column). Scale bar: 50 μm. **(C)** Plot of the mean E-cadherin immunofluorescence intensity against the depth from the bottom of tissues between CUBIC and CUBIC-BABB. *n* = 8. The error bars represent s.d. **(D)** Mean value of maximum intensity in each tissue between CUBIC and CUBIC-BABB. *n* = 8. P value > 0.05 for epididymis, kidney and lung (Welch’s *t* test). P values of less than 0.05 were considered to be statistically significant. The error bars represent s.d.

As shown in Fig 3A, we found that CUBIC and the PACT were superior to the other established methods in terms of fluorescence signal intensity, particularly in deeper regions of the tissues. Obviously, the signal intensity for the E-cadherin in each CUBIC and PACT treatment became more than twice that seen with other treatments at depths of 50–100 μm from the bottom of any organ (Fig 3A). However, the signal intensity in the lung suddenly decreased at depths greater than 50 μm. This decrease of signal intensity can be considered because the density of epithelial tubules that expresses the E-cadherin became lower in shifting to the proximal region. Significantly, CUBIC and PACT methods enabled us to observe the epididymal tubule at single-cell resolution even at depths of 300 μm interior to the organ (Fig 3B). This is also true of both the renal and lung tubules.

Instead of using a single established method, combining each process of the different clearing methods to optimize tissue clearance may allow more effective deep tissue imaging. Clearing methods that include steps for the removal of lipids, such as CUBIC or PACT, should effectively enhance the degree of antibody penetration into the tissue. However, the RIs of the solutions used in the CUBIC (1.49) and PACT (1.46) methods are less than the RI of the BABB solution (1.56), meaning that the mismatch of RI between the clearing solution and the biological tissues is greater with the solution used in the CUBIC or PACT methods than with that used in the BABB [10,12,13]. Hence, the process of lipid removal used in CUBIC or the PACT followed by tissue clearance by treatment with BABB may achieve clearer deep tissue imaging than when a single clearing method is applied.

To test this hypothesis, we combined protocol of CUBIC and that of BABB, i.e., removing lipids from the tissues with the CUBIC solution followed by clearing with the BABB solution (CUBIC-BABB), and examined the fluorescence intensity of labeled E-cadherin in each tissue. In the treatment of CUBIC-BABB, fluorescence signal intensity against the tissue depth was similar to one in the case of CUBIC treatment in any tissues (Fig 3C). Furthermore, there are no significant differences in the maximum fluorescence intensity on each sample between these treatments (Fig 3D), suggesting that the combinational clearing method did not remarkably improve deep tissue imaging in the murine developing epididymis (E18.5), kidney (E16.5), and lung (E15.5).

### Whole-Tissue Quantification through Digital Image Processing

In this section, we demonstrate automatic whole-tissue quantification for an ordinary cellular process, cell mitosis, obtained by 3D digital image processing. Here, we focus on the spatial distribution of mitotic cells in the epididymal tubule dissected at E15.5 and at E16.5 because it has been examined by counting over a stack of frozen sections [22]. Our aim in this section is to clearly show whether quantitative results obtained by whole-tissue quantification correspond to those obtained through the use of a different method, that is, thin sectioning of sliced preparations, by focusing on the particular organ.

**Fig 4:**
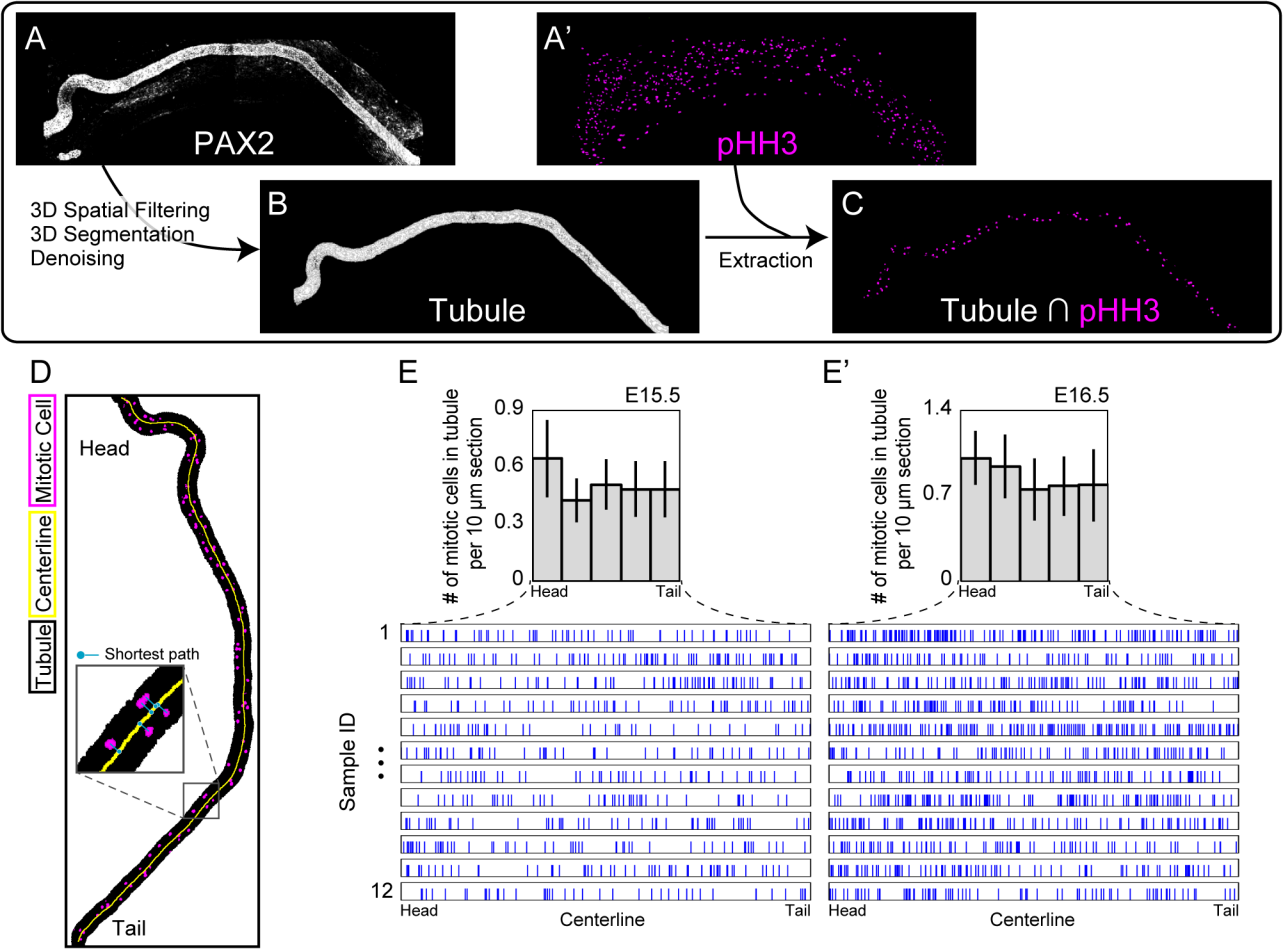
Whole-tissue quantification of the spatial distribution of mitotic cells along the epididymal tubule. **(A–C)** Overview of the procedure for digital image processing and 3D quantification used to acquire the spatial distribution of mitotic cells along the epididymal tubule. **(A, A′)** A projected raw image of the immunofluorescence of Pax2, a marker for the epididymal tubule, as well as that for phospho histone H3, a mitosis marker, in the epididymis dissected at E16.5. **(B)** The Pax2-positive cells were extracted from the image **(A)** as an epididymal tubule via 3D image processing. **(C)** Mitotic cells located only in the tubule were obtained by masking **(A′)** with **(B)**. **(D)** A superimposed image of the extracted epididymal tubule (black), extracted mitotic cells (magenta), and the centerline of the tubule was automatically obtained. The centerline was the result of image skeletonization **(B)**. A set of circles with lines colored in blue represent the shortest path to a position on the centerline from each mitotic cell. Note that an automatic count of mitotic cells along the tubule was performed in 3D, although the image was projected onto 2D for visualization. **(E, E′)** Spatial distribution of mitotic cells in the epididymal tubule along the centerline of the tubule at E15.5 and E16.5. The histogram integrates the raw data in which the position of the mitotic cell is represented as a pulse. The value on the vertical histogram axis was normalized to that in a 10-μm section. *n* = 12. The error bars represent s.d.

To extract cells undergoing mitosis only in the epididymal tubule, immunofluorescence signals from the mitotic cells scattering through the entire tissue were masked with those for the epididymal tubule. We regarded phospho histone H3 (pHH3)-positive cells to be mitotic cells and Pax2-positive cells as epithelial cells comprising the tubule in the epididymis (Fig 4A–4C; see the methods section for the details of digital image processing). Then, determining the centerline of the tubule using binary images of an extracted tubule and setting a point on the centerline connected to the centroid of a pHH3-positive object via the shortest path (Fig 4D), we can quantify the spatial distribution of mitotic cells along the tubule.

We examined the spatial distribution of pHH3-positive cells only in the epididymal tubule along its head-to-tail axis at each E15.5 and at E16.5 and found that the number of mitotic cells was greater in the head region than in the tail region (Fig 4E and 4E′). Indeed, the quantitative results obtained in the automatic detection of mitotic tubule cells in this study are almost consistent with the results obtained in an earlier study [22]. Note that the values on the vertical axis were normalized to be consistent with the previous report.

## Discussion

In this study, we have shown what each step of the entire process on the whole-tissue quantification including fixation for immunofluorescence, optical clearing, and digital image processing, bring to in the embryonic murine tubular organs such as epididymis, kidney, and lung. Throughout the present article, we exhibited representative results in each section by focusing on specific appropriate objects; however, it should be noted that these results are not necessarily applicable to any general situations. For example, in the section “Choice of Fixative Solutions”, we revealed that the TCA fixative gave better performance on the fluorescence labeling compared with the PFA fixative as exemplified by the pMLC immunostaining. However, this is not the case for phalloidin-labeling to the F-actin. As previously reported, phalloidin does not bind with the F-actin when the TCA fixation is used [23]; therefore, we could not simultaneously detect pMLC and F-actin labeled with phalloidin under the TCA fixation. We put emphasis that fixation conditions should be optimized specifically to what epitopes the investigators will observe.

The differences between the pMLC immunofluorescence results obtained with the two fixatives can be considered to arise from two causes. One is that the TCA fixative substantially inactivated the endogenous phosphatase, while the PFA fixative provided incomplete inactivation. This function of the TCA fixative was discussed in an earlier study, although their target epitopes are different from those employed in this study [23]. The other is that the TCA reduced the solubility of the pMLC epitopes by precipitation, leading to the appropriate fixation of antigen epitopes. It is unlikely that the former explains the improvement seen with TCA fixation because the overall pMLC signals were not significantly reduced even by PFA fixation (Fig 2). Therefore, it is reasonable to consider that the immobilization of pMLC epitopes caused by the TCA fixation allowed the clear detection of pMLC signals.

We expect that an integrated view of the non-uniform distribution of pMLC shown in Fig 2C with that of F-actin shown in Fig 1C would explain how the morphogenesis of the lung tubule proceeds. It has been clarified that pMLC regulates contractile activity by working with the F-actin, thus pMLC is known as a marker for active cellular constriction, giving rise to direct regulation of tissue deformation [24,25]. Taking these observations and knowledge into account, we believe that concentrated co-localizations of pMLC and F-actin in the proximal region of the lung tubule contribute to maintaining the radial size of the tubule by preventing tubule expansion due to the cell proliferation, while weak localization in the distal region aids the ampulla formation of the tubule that always occurs before the terminal branching [26,27].

Among the established optical clearing methods, the results shown in the Fig 3A suggest that CUBIC and PACT realized clearer imaging at greater depths than the other methods while maintaining appropriate resolution because these clearing methods include steps for lipid removal from tissues. These two methods eliminate lipid components in a different manner. In the process of CUBIC, the lipids are removed by simple immersion in a solution containing polyhydric alcohols [13]. In the PACT procedure, in contrast, lipids are rinsed with sodium dodecyl sulfate (SDS), and then the tissues are hybridized to hydrogel monomers to stabilize biomacromolecules [12]. Comparing the processes in these two methods, it can be emphasized that CUBIC uses a much simpler, lower-priced and much more convenient process of lipid removal from the tissues. In addition, applicability of the CUBIC method to various organs has been validated in this study, as well as demonstrated in an earlier study [14]. Taken together, we conclude that the CUBIC method is the most efficient method among established ones for comprehensive whole-tissue quantification when, in particular, high-throughput analysis is required.

As shown in the Fig 3C and 3D, the combined procedure of optical clearance including advantageous point of CUBIC and that of BABB did not achieve significant improvement in terms of clear deep imaging for the tissues, such as epididymis, kidney and lung. Indeed, these tissues contain less lipids in the embryonic stages we examined; therefore, rinse with CUBIC solution could hardly enhance antibody penetration into the deep region of tissues. As for applying to lipid-rich tissues such as brains, combinational approach of established optical clearing methods still has a possibility for better vivid volume imaging.

Regarding the last section of the Results, there are two major advantages of whole-tissue quantification, compared with the analysis by slice sectioning: objectivity and labor saving for the quantification. The whole-tissue analysis provides objective quantification because it can detect target molecules in the tissue without destroying the structural continuity of tissue, while classical quantification via slice sectioning of the tissues depends on individual techniques that often yield variable quantitative results. Therefore, whole-tissue analysis is a reliable method in terms of the reproducibility of quantification. In addition, whole-tissue quantification releases the analyst from time-consuming works because each process of imaging can be performed automatically, as can the digital image analysis.

## Materials and Methods

### Animals

Imprinting control region (ICR) mice were purchased from Japan SLC, Inc. We defined the plug date as embryonic day 0.5 (E0.5), and we used the embryonic tubular organs dissected at E13.5, E15.5, E16.5, and E18.5 for this study. The dissected kidney and lung were collected from embryos regardless of sex. The dissection was performed in accordance with earlier studies [28]. All the animal experiments were approved by the local Ethical Committee for Animal Experimentation of Institute for Frontier Medical Sciences, Kyoto University(authorization number: Z-3), and were performed in compliance with the Guide for the Care and Use of Laboratory Animals at Kyoto University. All mice were sacrificed by cervical dislocation to minimize suffering.

### Whole-Tissue Fluorescence Labeling

For whole-tissue labeling, samples were fixed with 4% PFA in PBS for 6 h at 4°C or with 2% TCA in Ca^2+^- and Mg^2+^-free PBS for 1 h at 4°C. These conditions were optimized for each fixative. In this study, we used 4% PFA/PBS as a fixative solution unless otherwise noted. The samples were then blocked by incubation in 10% normal goat serum diluted in 0.1% Triton X-100/PBS for 4 h at 37°C. The samples were subsequently treated with primary antibodies overnight at 4°C and then incubated with secondary antibodies conjugated to either the AlexaFluor 546 or the AlexaFluor 647 (1:1000, Invitrogen) overnight at 4°C. The following primary antibodies (1:200) were used: rabbit monoclonal anti-E-cadherin (Cell Signaling Technology), rabbit polyclonal anti-pMLC (Abcam), rabbit polyclonal anti-Pax2 (Invitrogen), and rat monoclonal anti-phospho-histone H3 (Abcam). We used TRITC-conjugated phalloidin (1:250, Millipore) and Hoechst 33342 (1:250, Invitrogen) for the visualization of F-actin and nuclei, respectively. Heat-induced epitope retrieval was skipped in this study; however, it may sometimes be required for better detection of target protein epitopes in whole-tissue immunofluorescence [29].

### Optical Tissue Clearing

In this study, CUBIC was applied to all samples unless otherwise described. For tissue clearing with BABB solution, we first immersed immunolabeled tissues in 50% (v/v) methanol/PBS, and stabilized the immunolabeled tissues on a glass-based dish (Iwaki) with 1% (w/v) agarose gel. The mounted tissues were dehydrated in 100% methanol. Then, the tissues were optically cleared with BABB solutions. Basically, we followed a standard protocol [8]. For treatment with SeeDB, we primarily referred to the original article [10]. Briefly, samples were first immersed in 40% (w/v) sucrose/PBS for 3 h at room temperature (RT), and then they were mounted on the dish. After incubation with 100% (w/v) sucrose/PBS for 3 h at RT, the samples were cleared with SeeDB for 8 hours at RT. For the CUBIC and PACT procedures, we first applied the steps for the removal of lipids before the whole-tissue immunofluorescence. For the CUBIC lipid removal, the tissues were collected in round-bottom 2ml tube (Watson) individually, and then, were immersed in CUBIC1 solution overnight at 4°C [13, 14]. Subsequently to the immunolabeling, the CUBIC2 solution was applied for optical clearing in the established CUBIC procedure. As for the CUBIC-BABB procedure, the tissues were cleared with the BABB solution as described earlier. For the PACT procedure, the tissues were collected in a 50ml centrifuge tube (Corning), and were immersed in 4% acrylamide in PBS (v/v) supplemented with 0.25% (w/v) VA-044 (Wako) overnight at 4°C. Then, they were incubated at 37°C for 2 h to initiate hydrogel hybridization in the tissues. After removing the hydrogel surrounding to the tissues, the lipids in the tissues were rinsed in 8% SDS in PBS (w/v) overnight at 37°C with gentle shaking [12].

### Microscopic Imaging

Samples were photographed using a confocal microscope (SP8, Leica) with objective lenses magnifications of ×20 (numerical aperture (NA) = 0.75, working distance (WD) = 680 μm, HC PL APO CS2, Leica), or ×40 (NA = 1.3, WD = 240 μm, Leica). We used 1% agarose gel to mount the samples on a glass-based dish (Iwaki) for stable imaging.

### Measurement of Mitotic Cells in the Epithelial Tubule

To automatically extract the images of mitotic cells only in the epithelial tubule of the epididymis, we used whole-tissue immunofluorescence images made using Pax2 as a marker for the tubule pHH3 as a marker for the mitotic cells. First, we performed median filtering on both the Pax2 and pHH3 images to remove noise, and then the Pax2 images were subjected to a 3D Gaussian filtering processed to make the edges of Pax2-positive pixels smooth. Next, the processed Pax2 and pHH3 images were transformed into binarized images using 3D maximum entropy thresholding [30]. Note that the method of thresholding should be chosen based on a histogram of the pixel intensities of the images [31]. In the binary Pax2 images, we regarded the connected component in 3D having the maximum number among all connected components as representing the tubule; we call this stack of images the tubule images. Then, masking the binarized pHH3 images by exploiting the tubule images, we extracted the mitotic cells only in the tubule. Finally, determining the centerline of the tubule by skeltonization, the position of mitotic cells along the tubule was calculated by taking the shortest path from the centroid of each mitotic cell to the centerline. Image processing and quantification was performed with MATLAB software (MathWorks). The MATLAB script files are available with an image file we used in the analysis in supporting information files (S1 File).

## Supporting Information

S1 File Compressed zip file including MATLAB script codes and a raw image file we used for the analysis of Fig 4. (ZIP)
